# Prognostic role of C-reactive protein in patients with nasopharyngeal carcinoma: A meta-analysis and literature review

**DOI:** 10.1097/MD.0000000000008463

**Published:** 2017-11-10

**Authors:** Yi Fang, Chang Xu, Peng Wu, Ling-Hao Zhang, Da-Wei Li, Jie-Hao Sun, Wen-Feng Li, Zhi-Su Liao

**Affiliations:** a Department of Otorhinolaryngology, The First Affiliated Hospital of Wenzhou Medical University; b Wenzhou Medical University; c Department of Anesthesiology, The First Affiliated Hospital of Wenzhou Medical University; d Department of Radiochemotherapy, The First Affiliated Hospital of Wenzhou Medical University, Wenzhou, Zhejiang, China.

**Keywords:** C-reactive protein, meta-analysis, nasopharyngeal carcinoma, prognosis

## Abstract

**Background:**

C-reactive protein (CRP) has been shown to be associated with several tumors. However, its association with nasopharyngeal carcinoma (NPC) is not well characterized. We performed a literature review and meta-analysis to assess the prognostic relevance of elevated CRP levels in patients with NPC.

**Methods:**

A literature search for relevant studies was performed on PubMed (Medline), the Cochrane Library, and Web of Science databases. Hazard ratios (95% confidence intervals) were calculated to assess the association between elevated CRP levels and survival outcomes.

**Results:**

Five studies with a combined study population of 5215 patients with NPC were included. Pooled hazard ratios for overall survival and distant metastasis-free survival were 1.84 (95% CI = 1.57–2.17) and 1.81 (95% CI = 1.53–2.14), respectively. Subgroup analyses showed that types of indicators and treatment before inclusion had no significant impact on the observed association.

**Conclusion:**

Elevated serum CRP levels in patients with NPC were associated with worse prognosis.

## 1 Introduction

Nasopharyngeal carcinoma (NPC) is a classical head and neck cancer with a skewed global distribution. The associated morbidity rate in southern China and South Asia is of the order of 20 to 30 per 100,000 population.^[1]^ NPC typically originates from the epithelial lining of the nasopharynx; the most common histotype is squamous-cell carcinoma. Currently, the standard treatment modalities for NPC are chemotherapy and radiotherapy.^[2]^ Despite advances in treatment of NPC, incidence of recurrence within a few years of treatment completion continues to be a challenge.^[3,4]^ Identification of new risk-stratification indices may help optimize treatment strategy for patients with NPC.

C-reactive protein (CRP) is a sensitive marker of systemic inflammation. As a component of the innate immune system, CRP can recognize membrane constituents of damaged cells as well as some foreign pathogens.^[5]^ Owing to its superior temporal stability and the availability of reliable assays, CRP is a particularly suitable inflammatory marker for prognostic stratification in clinical settings.^[6]^ Although a link between inflammation (CRP) and cancer was first proposed by Virchow in 1863, it was only in the last 2 decades that we began to focus on the intricate network of interactions.^[7]^ The significance of CRP levels as a clinical predictor of survival has been demonstrated in the context of gastrointestinal,^[8]^ breast,^[9]^ renal,^[10]^ ovarian,^[11]^ lung,^[12]^ and hepatocellular carcinoma.^[13]^ However, due to a paucity of data on the relationship between CRP levels and risk of NPC, the prognostic role of CRP levels in patients with NPC is not clear.

Hence, we performed a meta-analysis and literature review to explore the association between serum CRP levels and survival of patients with NPC. Pooled data from studies that investigated the association of CRP levels with overall survival (OS) and distant metastasis-free survival (DMFS) were analyzed.

## 2 Materials and methods

### 2.1 Search strategy and selection criteria

We searched PubMed (Medline), the Cochrane Central Search library, and Web of Science databases for published studies that analyzed the effects of CRP in patients with NPC up to December 31, 2016. The keywords used for literature search were: “C-reactive protein” or “CRP” combined with “nasopharyngeal carcinoma” or “nasopharyngeal cancer” or “NPC.” No language limits were applied. Only human studies were included. Abstracts of all candidate articles were reviewed by 2 independent reviewers (YF and CX). Any disagreements were solved by further discussion by the review team. The project was following ethical and institutional guidelines and was approved by the Medical Ethics Committee of First Affiliated Hospital of Wenzhou Medical University (Registration number: 2017037).

Primary studies that met the following criteria were included: pretreatment CRP levels measured with use of serum-based methods; histopathological diagnosis of NPC; correlation of CRP with OS, DMFS, or cancer-specific survival (CSS); and availability of adequate data to calculate hazard ratios (HRs) and 95% confidence intervals (CIs). Studies that did not report HRs were included if adequate data was available to calculate HRs. Primary studies with the following characteristics were excluded: Duplicate data or repeat analysis—if several studies were published by the same group with overlapping patient populations, the most recent article with better range of information was chosen to avoid data duplication; studies with sample size of <20 patients; and case reports, meeting records, and the review articles.

### 2.2 Quality assessment and data extraction

We used the PRISMA checklist (available online at http://www.prisma-statement.org/) to assess whether all components required for a meta-analysis were included in this study. Quality assessment of all studies included in the meta-analysis was performed according to The Newcastle-Ottawa Scale (NOS).^[14]^ Data pertaining to the following variables were extracted from the included studies: name of first author, publication year, duration of follow-up, number and classification of patients, pre-inclusion treatment, therapeutic methods, cutoff values used to determine pathological elevation of CRP level, technique used for measurement of CRP level, type of survival outcomes reported, and HRs with their 95% CIs.

All statistical analyses were performed with Stata software package (version 12, Stata Corp, College Station, TX). A 2-tailed *P* <.05 was used as the criteria for statistical significance. Pooled HRs and 95% CIs were calculated to assess the association between CRP levels and OS or DMFS. A combined HR >1 with nonoverlapping 95% CI was considered indicative of a statistically significant positive association with a worse OS or DMFS. Chi-squared test and *I*^2^ were used to evaluate the heterogeneity between the included studies.^[15]^*I*^2^ >50%, was considered indicative of significant heterogeneity, and random-effects model was used for the analysis. If not, a fixed-effect model was used. Sensitivity analysis was performed to assess for potential bias across the studies. Subgroup analyses were conducted according to the type of marker as well as pre-inclusion treatment. Publication bias was assessed by means of Begg plots, along with Funnel plot, and Egger tests.

## 3 Results

**Figure 1:**
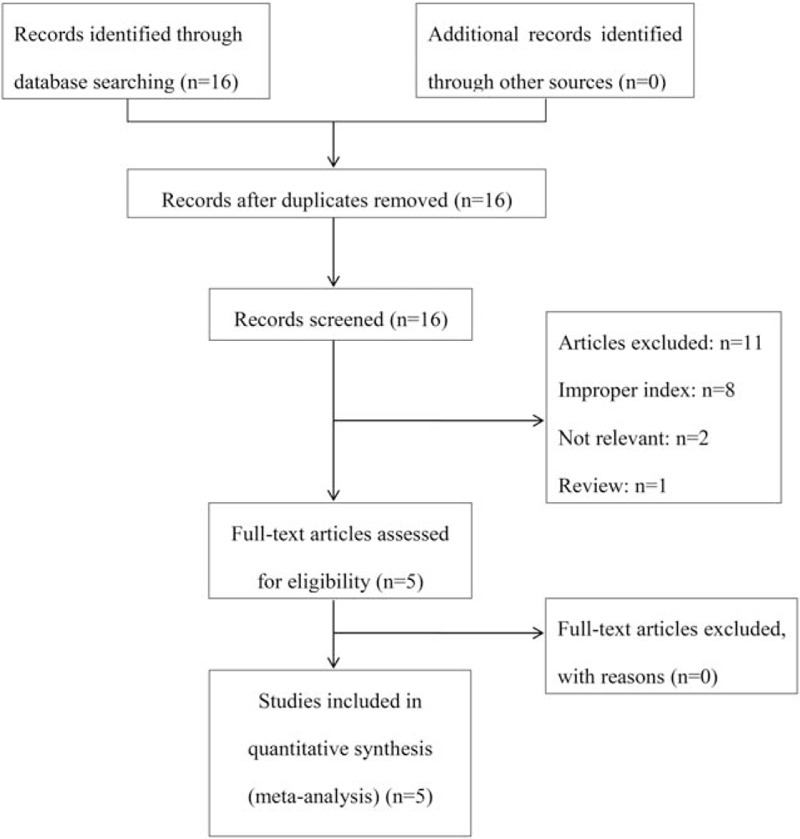
Schematic illustration of the literature search and study-selection criteria.

A total of 16 studies were retrieved on initial literature search. Of these 11 studies^[16–26]^ were excluded after review. The search results are shown in Figure 1. Finally, 5 studies^[27–31]^ with a combined study population of 5215 patients with NPC were included in the analysis. The characteristics of the 5 eligible studies are summarized in Table 1. It should be pointed out that the study by Zeng et al^[31]^ included in the meta-analysis reported CSS. Despite the difference from other studies which reported OS or DMFS, the statistical measures described in this study were comparable with those in the other 4 studies. Moreover, data for calculation of OS were also available (Fig. 1). Therefore, we merged the data with those of other studies. There was no overlapping of study population among the 5 studies. The NOS scores of studies ranged from 4 to 7 (mean: 6).

**Table 1:**
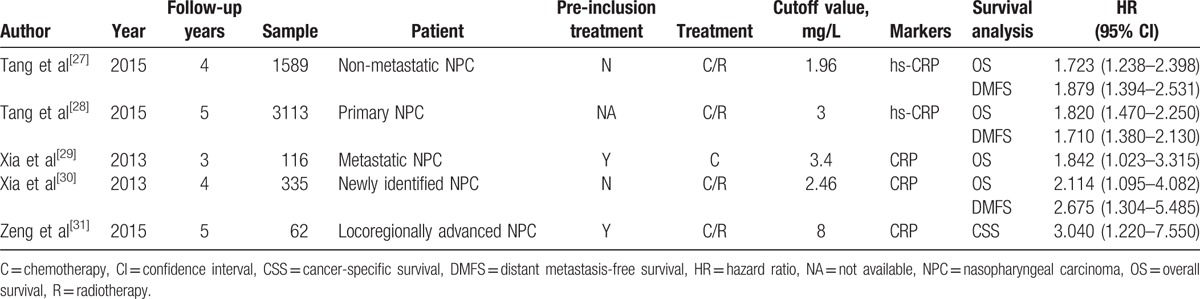
Characteristics of studies included in the meta-analysis.

**Figure 2:**
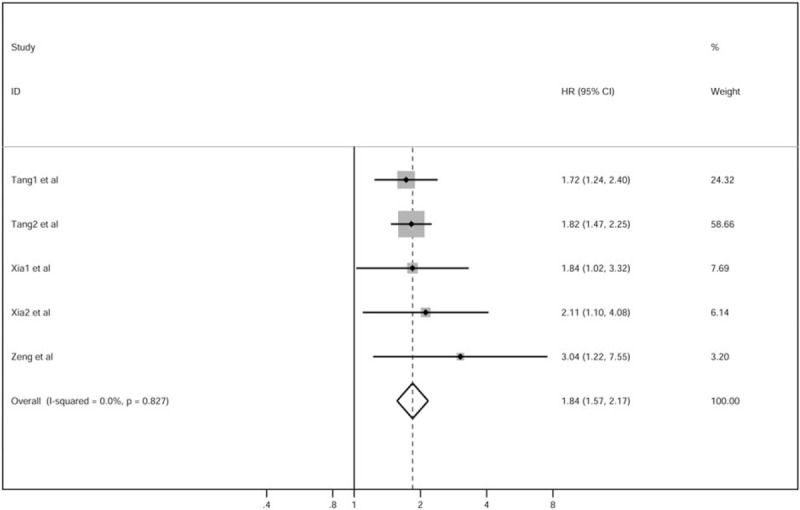
HRs for OS with high CRP. *P* values based on the Cochrane Q test for heterogeneity. CI = confidence interval, CRP = C-reactive protein, HR = hazard ratio, OS = overall survival.

**Figure 3:**
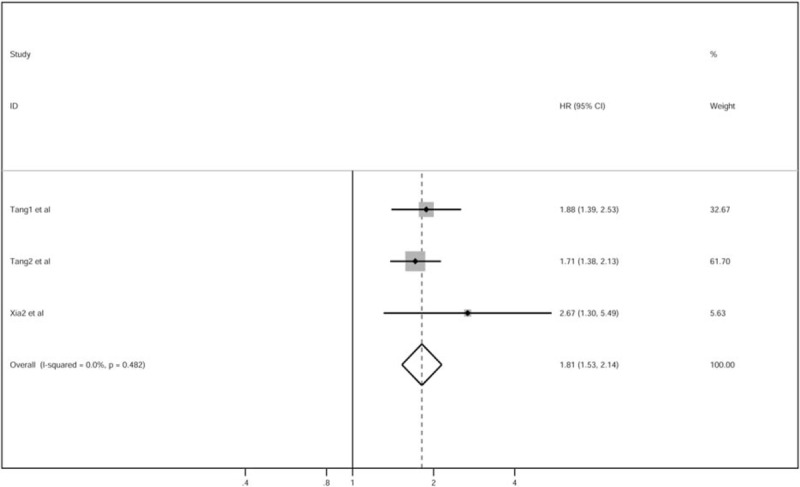
HRs for DMFS with high CRP. *P* values based on the Cochrane Q test for heterogeneity. CI = confidence interval, CRP = C-reactive protein, DMFS = distant metastasis-free survival, HR = hazard ratio.

Pooled analysis of data from the included studies showed an association between elevated CRP levels and OS and DMFS in patients with NPC. On multivariate analyses, adjusted HRs (95% CI) for OS and DMFS were 1.84 (1.57–2.17) and 1.81 (1.53–2.14), respectively (Figs. 2 and 3). Based on the receiver operating characteristic (ROC) curve analysis, the cutoff values were found to be close to 3 mg/L. The number needed to treat (NNT) was 3968. Despite differences with respect to patient-selection methods between the included studies, the pooled analysis still yielded homogeneous effects (*I*^2^ [*P*] 0% [.827] for OS, *I*^2^ [*P*] 0% [.482] for DMFS). On subgroup analyses based on marker and pre-inclusion treatment, the association between high levels of serum CRP and OS was still significant (Table 2).

**Table 2:**
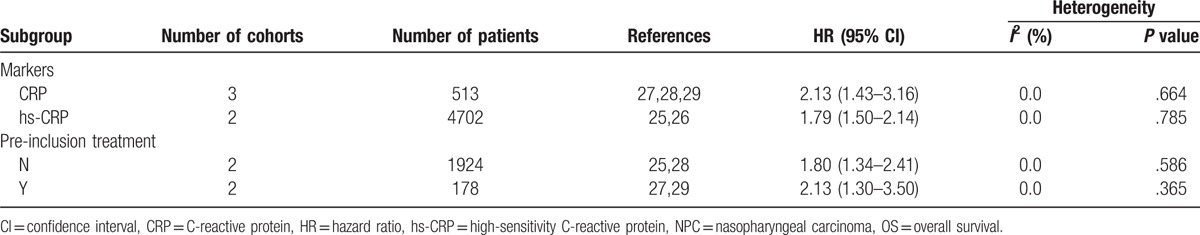
Subgroup analyses of pooled HRs for increased serum CRP and OS of patients with NPC.

To detect potential influence of each of the 5 included studies on the pooled results, sensitivity analysis was performed by sequential omission of one study at a time. The results showed that the observed association was not significantly affected by any one particular study (data not shown). The results demonstrated the stability and robustness of our analysis.

**Figure 4:**
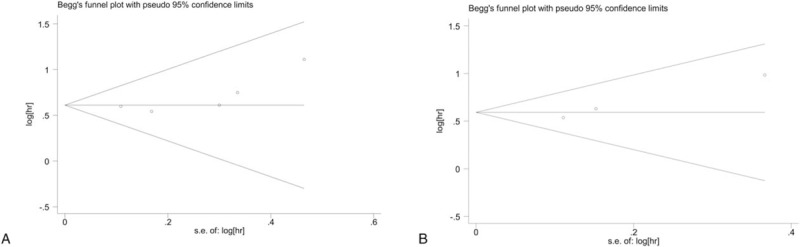
Begg funnel plots for publication bias testing (A) OS and (B) DMFS. DMFS = distant metastasis-free survival, OS = overall survival.

Begg test, along with funnel plot and Egger tests did not show any significant publication bias (Fig. 4). However, the possibility of potential publication bias could not be entirely ruled out due to the limited number of the included studies. Moreover, all studies supported the significant relationship between CRP and poor outcomes of patients with NPC.

## 4 Discussion

In this study, we sought to demonstrate the relationship between CRP levels and prognosis of patients with NPC. Overall, our results support the hypothesis that high serum levels of CRP in patients with NPC are associated with poor prognosis. There was a significant homogeneity between the included studies with respect to the meta-analysis for OS. Sensitivity analysis further confirmed the consistency of the results.

The association was found to be statistically significant on subgroup analysis after stratification of data by type of marker and pre-inclusion treatment. In other words, the type of marker and pre-inclusion treatment had no significant impact on the observed association between high CRP levels and survival outcomes of patients with NPC.

Inflammation is known as the seventh hallmark for tumor formation and development.^[32]^ Inflammation acts as an intrinsic (immune-mediated inflammation and tumor microenvironment) as well as an extrinsic factor in carcinogenesis.^[33]^ In clinical practice, low levels of cytokines and growth factors are used as supportive indices of therapeutic efficacy. High levels were associated with unfavorable prognosis in cancer, such as NPC.^[34,35]^

CRP is a nonspecific protein present in acute-phase inflammation and also reacts to infection and tissue injury. During acute response, cytokines, predominantly IL-6, are secreted from damaged tissue and promote the synthesis of CRP in the liver.^[36]^ Even though CRP was identified 80 years ago^[37]^ and has been used as a valuable inflammatory marker, research on the relationship between inflammation and carcinogenesis gained momentum only in the last 2 decades.^[38]^ For instance, the Glasgow Prognostic Score (GPS), a simple inflammation-based score based on serum albumin and CRP levels, was shown to be useful in predicting survival of patients with breast, pancreatic, ovarian, and non-small cell lung cancers.^[39–42]^ Another prognostic scoring model (TNM-C model) based on serum CRP and the classical TNM-classification has been used to estimate the risk of death among patients with clear cell renal cell carcinoma and in particular, to identify patients likely to benefit the most from adjuvant therapy.^[43]^

The causative relationship between CRP and cancer has not been investigated since variations in CRP levels are not specific to cancers.^[44]^ In the context of NPC, high levels of CRP could be a consequence of obstruction of the nasal cavity by the cancerous mass, which causes inflammation in the paranasal sinuses. Since much of the available evidence emanates from small-scale retrospective studies, our meta-analysis adds value to the current literature by presenting a synthesis of the current evidence. Our findings confirm that serum CRP may serve as a prognostic indicator in patients with NPC.

The reasons that serum CRP level is relevant to oncological outcomes including NPC are not clear. The hypothesis is as follows: First, high levels of CRP can be due to host reactions to nonspecific infection or local tissue injury or tumor necrosis.^[45,46]^ Second, tumor cells are always in a state of chronic inflammation (also referred to as the tumor microenvironment).^[47]^ Chronic phlogosis makes the microenvironment conducive for carcinogenesis and development, which can specifically induce DNA damage, cancer-derived angiogenesis, and distant metastasis.^[48]^ Third, inflammatory factors, such as interleukin-6 (IL-6) and IL-8, participate in oncogenesis by acting directly on normal cells via signaling pathway such as NF-κB1, which also induce hepatocytes to produce excessive CRP.^[49]^

The present study has several advantages: First, a large cluster of samples was included in the study. Moreover, the statistical results were not unduly influenced by any one particular study. Further, the lack of heterogeneity among the included studies is strength of the study.

Notably, several limitations exist in our meta-analysis: First, the studies included in this meta-analysis showed much heterogeneity with respect to duration of follow-up and treatment, which could have had an impact on the observed association. Second, all the studies were from China. Among the 5 included studies, Tang was the author of 2 papers^[27,28]^ and so was Xia.^[29,30]^ Even though the studies pertained to a limited region, this largely reflects a greater research focus on the Chinese population owing to the vulnerability of Chinese people.

In conclusion, our meta-analysis suggests that elevated serum levels of CRP denote a worse prognosis of patients with NPC. Further research including large prospective studies is required to draw more definitive conclusions.

## Acknowledgments

We thank Wei-Qing Fang, Hui-Mei Li, Ya-Mei Luo, and Hui-Wen Xiao for their review and comments on the manuscript.
